# Taming Parasites by Tailoring Them

**DOI:** 10.3389/fcimb.2017.00292

**Published:** 2017-07-06

**Authors:** Bingjian Ren, Nishith Gupta

**Affiliations:** Faculty of Life Sciences, Institute of Biology, Humboldt UniversityBerlin, Germany

**Keywords:** parasite manipulation, CRISPR, genetic engineering, protozoan infections, genome editing

## Abstract

The next-generation gene editing based on CRISPR (clustered regularly interspaced short palindromic repeats) has been successfully implemented in a wide range of organisms including some protozoan parasites. However, application of such a versatile game-changing technology in molecular parasitology remains fairly underexplored. Here, we briefly introduce *state-of-the-art* in human and mouse research and usher new directions to drive the parasitology research in the years to come. In precise, we outline contemporary ways to embolden existing apicomplexan and kinetoplastid parasite models by commissioning front-line gene-tailoring methods, and illustrate how we can break the enduring gridlock of gene manipulation in non-model parasitic protists to tackle intriguing questions that remain long unresolved otherwise. We show how a judicious solicitation of the CRISPR technology can eventually balance out the two facets of pathogen-host interplay.

## Living off the host for their entire life—the parasitic protists

Our acquaintance with parasitic infections and primary culprits dates back to ancient time (1500 BC), as evident in the Egyptian and Greek literature and through discoveries of parasites in archeological expeditions (Cox, 2002). The first major technological revolution spurring the study of protozoan parasites, the focus of this article, was the invention of microscope and its prudent deployment by Antonie van Leeuwenhoek in seventeenth century (Lane, 2015). A systematic learning of the discipline however did not commence until confirmation of the germ theory two centuries later following the pioneering work of Pasteur and his colleagues and sharp dismissal of the long-overdue notion of spontaneous generation (Smith, 2012). Afterwards, basic research on the parasitic protists has ensued along two reciprocal lines: finding a parasite and recognizing its subsequent relationship to the disease, or conversely by distinguishing a disease and then discovering the causative offender. The second methodical innovation was our ability to culture parasites, albeit only few thus far, which steered the field for follow-up breakthroughs, namely genetic engineering and omics. In this perspective review, we will discuss how the latest technological expansion, the CRISPR system, is poised to transform the discipline of molecular parasitology in a way that was just not feasible earlier.

**Figure 1:**
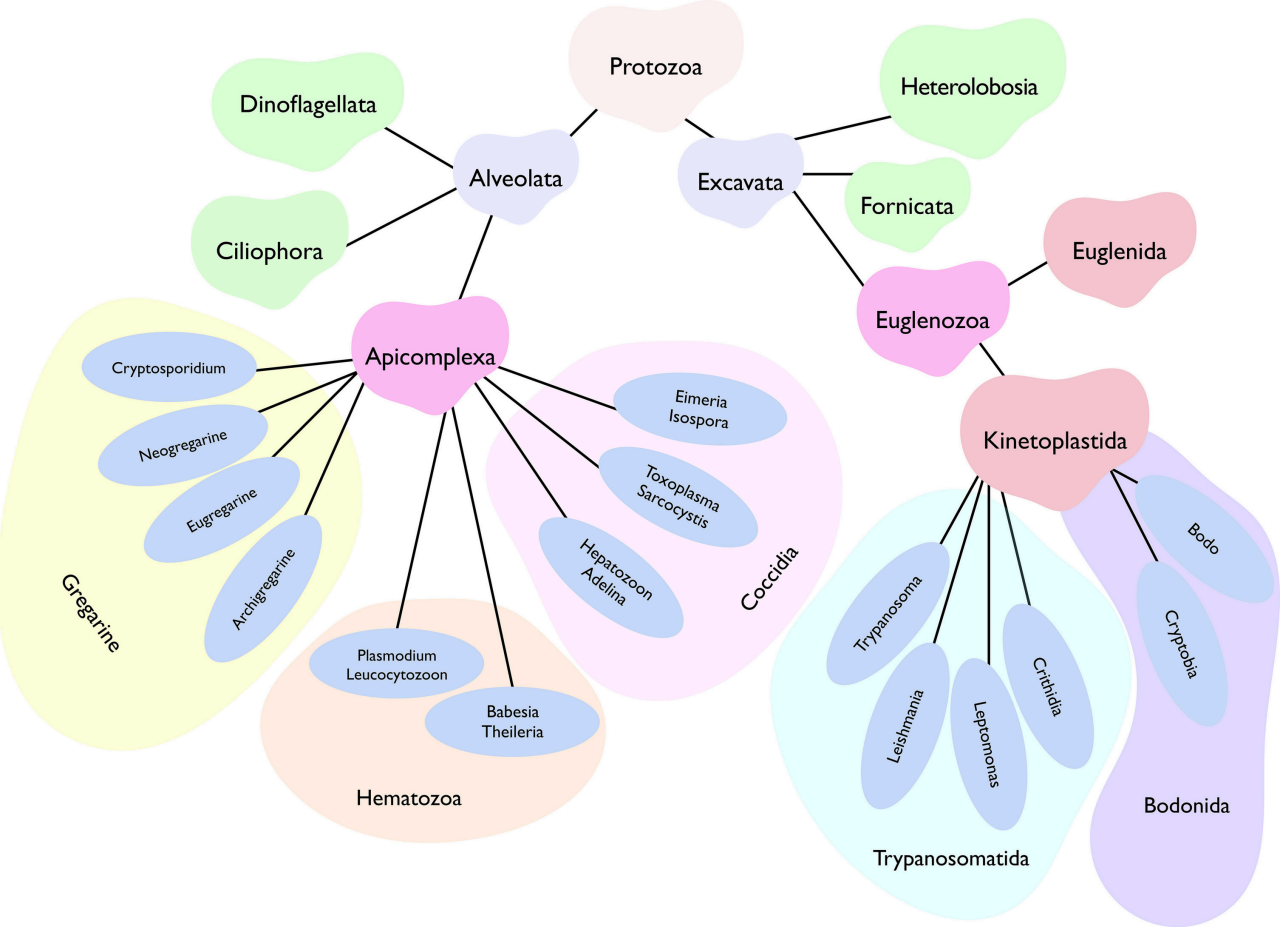
Abridged phylogenetic tree depicting main super-phyla of the kingdom protozoa, namely Alveolata and Excavata. Two of the all shown phyla, Apicomplexa, and Kinetoplastida, comprise a vast majority of human and animal pathogens. Only the selected genera representing each class are displayed. While most apicomplexans (except for gregarines) favor an intracellular lifestyle, kinetoplastids prefer an extracellular life (barring certain stages of *T. cruzi* and *Leishmania*). Besides, apicomplexan parasites exhibit well-defined asexual as well as sexual reproduction, whereas the latter phase is not yet known in most kinetoplastids. Individual genera or even species have evolved a notably distinct lifecycle in specific host organisms, which often involves a perpetual inter-host transmission in nature.

The kingdom protozoa comprise more than 40,000 known single-cell extant species, of which about 25,000 occur as free-living, while the remaining have adapted to a parasitic lifestyle (Adl et al., 2007). The latter group includes at least 6,000 apicomplexan, 2,500 ciliate, 1,800 flagellate, and 250 amoebae species. These eukaryotic pathogens have acquired countless niches dispersed across the tree of life. Just humans and livestock alone serve as hosts to a startling number of parasites with many protists among them. Parasites belonging to two phyla, namely apicomplexa and kinetoplastida, account for a majority of infections (Figure 1). *Plasmodium, Toxoplasma, Eimeria, Sarcocystis, Cryptosporidium, Theileria, Babesia, Trypanosoma, Leishmania* and *Cryptobia* are some of the notorious genera to name but a few. Then there are many other rather unappreciated genera, whose recognition is limited merely to the taxonomy books (Levine, 1961). Collectively, all these parasites impose a significant burden on human and animal healthcare as well as on food industry. Not only do they affect the infected hosts by altering growth, behavior, nutritional status, reproductive abilities and mortality, but also shape our ecosystem by swaying trophic interactions, food webs and biodiversity (Torgerson et al., 2015; Cable et al., 2017).

**Figure 2:**
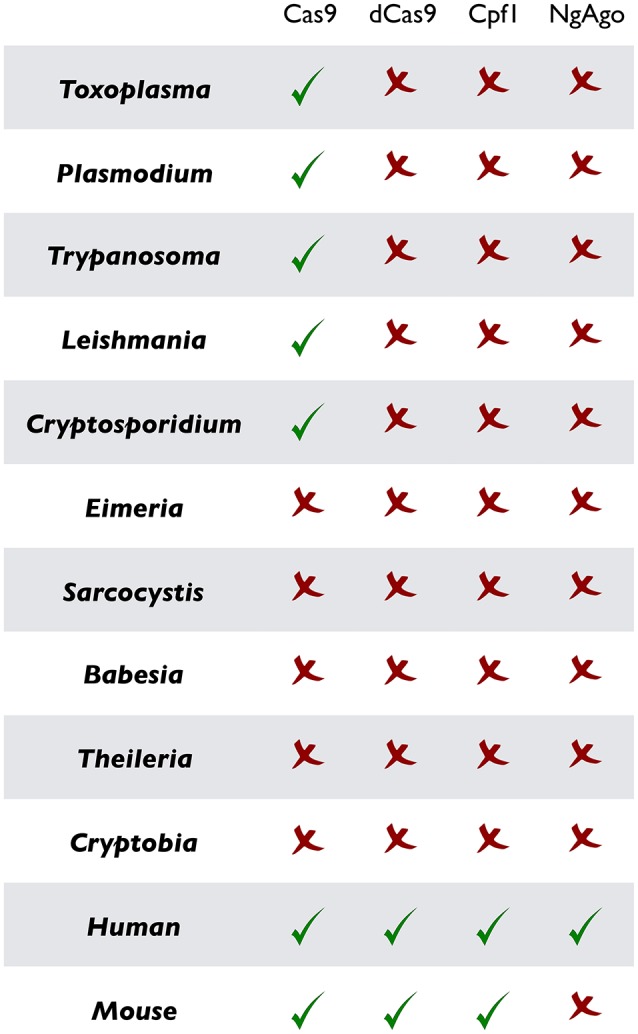
Current implementation of CRISPR or CRISPR-like tools in prototypical parasitic protists and their mammalian hosts. As noted, CRISPR/Cas9 and ensuing methods (dCas9, Cpf1, and NgAgo) have been successfully established in the mammalian cells, but remain widely marginalized in parasitology. Only the original CRISPR/Cas9 in designated parasites has been used so far.

**Figure 3:**
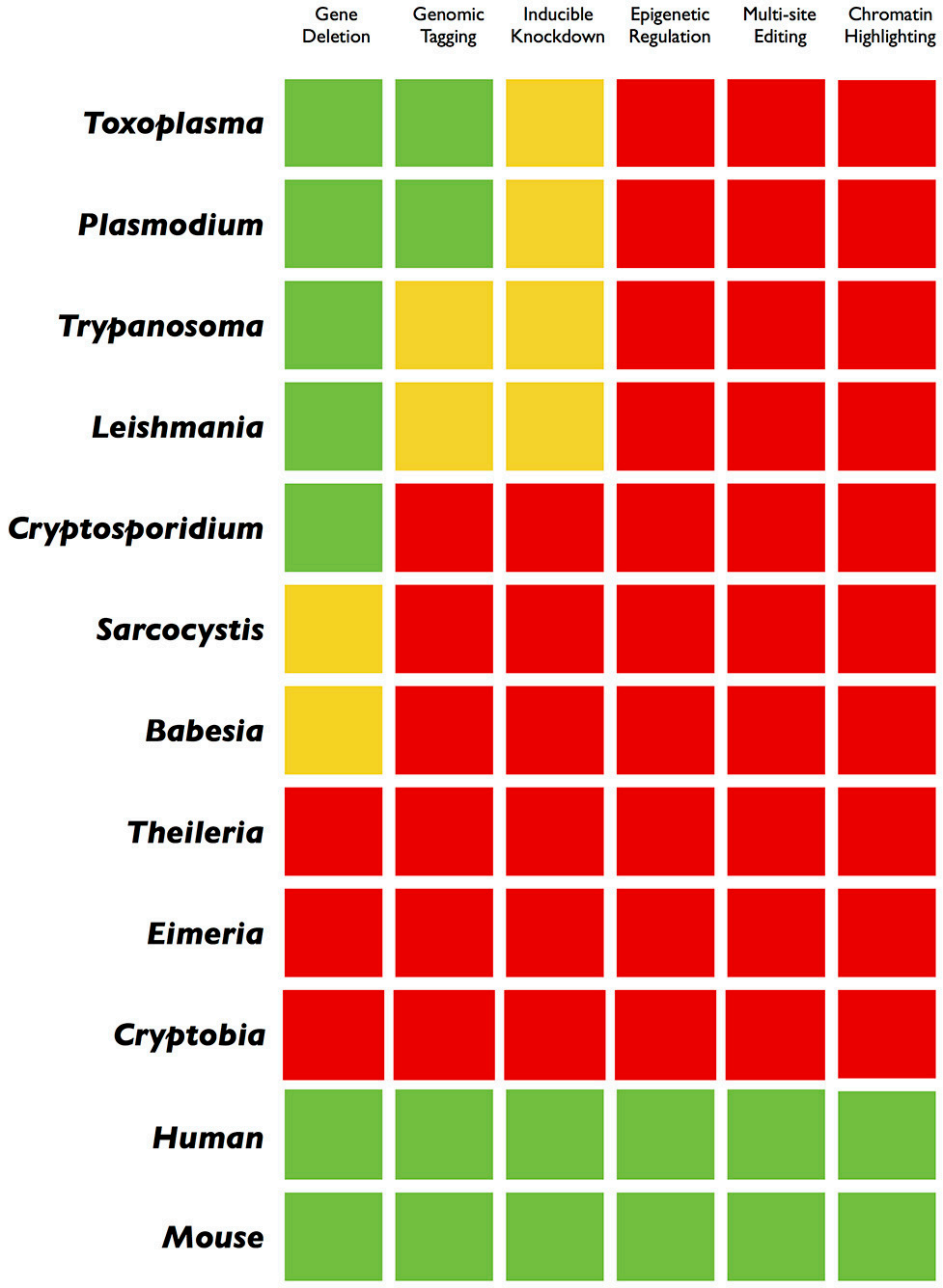
Traffic light gradient depiction of genome engineering applications in parasites and mammalian cells. A comparative color-coding indicates the current progress in different organisms. The green and red colors display a “comprehensive” or “not-at-all” scenario, respectively. The yellow light reflects an incremental success achieved through customary methods. While the green and yellow colors in model parasites encourage for more innovative applications (inducible gene silencing, epigenetic/epigenomic studies, multi-site editing), the widespread red color, mostly in non-model pathogens, advocates for a systematic application of the CRISPR technology.

Besides clinical, socioeconomic, and ecological relevance, the unicellular eukaryotic pathogens bestow a matchless opportunity to resolve appealing biological paradigms that have fascinated microbiologists since their discoveries. The natural lifecycle of parasites often gyrates between the primary and secondary hosts fostering asexual and sometimes sexual development, which is somewhat similar to multicellular counterparts but intriguingly genetically wired in a single cell. Moreover, because parasites depend on hosts, they offer a unique possibility to learn how two non-mutual symbiotic entities interact with each other. Many of them develop within a target host cell (i.e., a eukaryotic cell within a eukaryotic cell), a process that abstractly parallels intracellular bacterial pathogens, but mechanistically differs in host-pathogen Armageddon. Not least, it is even more thought provoking to envision the singularity of interactions required by each parasite to co-opt a specific host. Thus, research on as many representative parasites as plausible is crucial to gain a holistic insight into the concept of living together. Ironically however, only a handful of them have attracted the attention, mainly because most others are not amenable to *in vitro* culture or genetic manipulation. While the former issue can be partially circumvented by *in vivo* infection, gene tractability is still in its infancy for most parasites, when comparing to what has been achieved in mammalian models (Figures 2, 3). Given the progress made in recent years, specifically the CRISPR technology, we believe that it is now the time to upgrade and empower parasites to level the field with their mammalian hosts.

## An ultimate scissor to tailor any genome—the CRISPR system

CRISPR-based genome manipulation has originated from certain bacteria, which deploy it as a defense strategy to fend off viral infections (Koonin and Makarova, 2013). The system in its simplest form consists of two components, a short guide RNA (gRNA) and an endonuclease of the Cas family with a high affinity to the guide sequence. The simplicity of the tool has enabled CRISPR/Cas9 as one of the most powerful gene-editing tools ever. Its easy-to-design feature along with high specificity, efficiency, and low-cost have obsoleted other hitherto-popular genetic engineering methods, e.g., Zinc-finger and TALEN, which deploy custom-made peptides targeting the desired gene sequence (Gaj et al., 2013). The CRISPR system employs expression of small gRNA recognizing the gene of interest. Further, the use of gRNA enables large-scale gene editing by transfecting simply one or two plasmids, whereas Zing-finger and TALEN-based techniques are difficult to upscale due to challenging ectopic expression of multiple customized proteins in a single cell.

A comprehensive mechanistic insight into CRISPR-mediated genetic manipulation can be found in an excellent review by Sander and Joung (2014). In brief, the elementary application of CRISPR/Cas9 involves non-homologous end joining (NHEJ) and homology-directed repair (HDR). For NHEJ, a plasmid-construct expressing the Cas9 and gRNA is required, which create a nick at the intended locus followed by mutation-prone self-repair of the genomic DNA. A main issue encountered with the NHEJ-CRISPR/Cas9 gene editing is the lack of a selection marker, thus making it impractical to isolate mutants. This can however be avoided by HDR-CRISPR/Cas9 approach that requires transfection of an apposite CRISPR/Cas9 plasmid together with a donor construct comprising homologous fragments and drug selection cassette. Upon CRISPR/Cas9-mediated cut, the donor DNA recombines with the target locus leading to a precise editing and insertion of the selection marker. When compared to classical approaches, a gRNA-guided Cas9 cleavage in the genome improves the efficiency of transgenic work in an exponential manner. Not surprisingly, the system has been applied to a repertory of organisms (Sander and Joung, 2014), including selected parasites, as described below. Indeed, its unrivaled success has also made the high-throughput editing a reality in less than a decade.

## Enrich the rich but don't damn the poor—model vs. non-model

From a technological perspective, parasites can be categorized as either model or non-model parasites. *Toxoplasma, Plasmodium, Trypanosoma*, and *Leishmania* are the most well-studied genera, which have witnessed a progressive advancement in genome engineering, particularly in the last two decades (Teixeira and daRocha, 2003; de Koning-Ward et al., 2015; Reinke and Troemel, 2015; Wang et al., 2016). However, a considerable gap still exists between what we have achieved vs. where we want to be. The current transgenic approaches in model parasites are limited to ectopic expression, genetic knockout, genomic tagging, and conditional mutagenesis (Figure 3). Even execution of these strategies often relies on conventional tools, which makes the gene editing a daunting, time consuming, and pricy task. In certain fastidious species like *Plasmodium*, which are demanding to culture, the entire approach is further complicated (de Koning-Ward et al., 2015). It may take weeks to months to establish a transgenic line for downstream analysis, and failure in construction of mutants are mostly detected very late. Nonetheless, these parasite models have taught us (and continue to do so) a great deal about parasitism as a common mode of life. In particular, they have proven indispensable to appreciate the asexual reproduction and underlying interactions with hosts, which is crucial in our fight to eliminate the disease they cause.

A yet another grand challenge to overcome is to perform any sort of tangible manipulation to study gene functions in non-model organisms. Some of them have been minimally engineered and the road to their wide-ranging manipulation is long and treacherous (Clark et al., 2008; Suarez and McElwain, 2010; Dangoudoubiyam et al., 2014; De Goeyse et al., 2015; Vinayak et al., 2015; Figure 3). In this regard, *Cryptosporidium, Eimeria, Sarcocystis, Babesia, Theileria*, and *Cryptobia* species are fascinating because they are set to provide complementary biological insights. For instance, *Cryptosporidium* with <4000 genes has a highly abbreviated genome, and inhabits an extra-cytosolic (epicellular) vacuole as opposed to intracellular residence of mainstream apicomplexans (Lendner and Daugschies, 2014). *Eimeria* species completing their lifecycle in one host can illuminate the sexual development of coccidians (Walker et al., 2013), which remains heavily understudied (Smith et al., 2002). Equally, *Sarcocystis* resides freely in the host cytosol (Fayer, 2004), *Babesia* mimics many features of cerebral malaria in humans (Krause et al., 2007) and *Theileria* exerts a cancer-like phenotype to infected lymphocytes (Tretina et al., 2015); these apicomplexans have therefore potential to reveal exclusive developmental aspects (Striepen et al., 2007; Plattner and Soldati-Favre, 2008). Along the line, *Cryptobia* species exhibit an ectoparasitic phase in the lifecycle (Woo, 1987; a rarity among endoparasites), which may shed light onto evolution of distinct lifestyles. Not least, there are countless other parasites that may or may not be relevant from a clinical or veterinary stance but exhibit idiosyncratic lifestyles, which makes the biology of parasitism an attractive discipline for many of us. In the ensuing text, we outline numerous ways how we can exploit the power of CRISPR-based methods to further advance the current models, and more importantly, to break the genetic bottleneck in non-model parasites.

## Crunching hard with CRISPR—tailoring the parasite genomes

**Figure 4:**
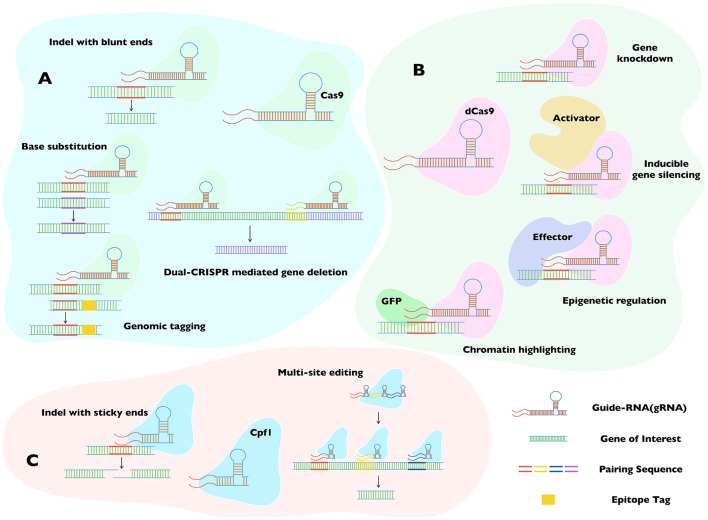
Major applications of Cas9, dCas9, and Cpf1 in genome editing. The three widely accepted CRISPR-dependent systems **(A–C)** broadly complement each other, even though the specified usage are not always restricted to individual proteins, i.e., Cas9 **(A)** and Cpf1 **(C)** can substitute each other to perform at least some of the mentioned tasks. Both enzymes require unique protospacer adjacent motif (PAM) to locate the gene of interest, making them complementary when no proper PAM is available for any of them. RNA-guided double-strand break is vital to their functioning. Whereas, Cas9 incision generates blunt ends, Cpf1 produces cohesive ends. The dCas9 **(B)** method can recompense for Cas9, when the target genomic loci happen to be essential. The inactivated isoform of Cas9 serves as a blocker, enabling a silencing of gene transcription instead of disrupting the gene locus. Its low toxicity also allows the assembly of stable progenitor lines for successive transgenic research. Not least, the feature that dCas9 can bind harmlessly to a locus of interest permits a fusion of dCas9 with reporter or effector proteins, further expanding its utility to epigenetic and epigenomic studies. Cas9, CRISPR associated protein 9; Cpf1, Centromere and Promoter Factor 1; dCas9, catalytically dead Cas9; cr-RNA, CRISPR-RNA; Indel, insert or deletion (of bases in the genome).

Initial application of CRISPR/Cas9 has already been achieved in *Toxoplasma, Plasmodium, Trypanosoma*, and *Leishmania* species (Ghorbal et al., 2014; Lee and Fidock, 2014; Peng et al., 2014; Shen et al., 2014; Sidik et al., 2014; Wagner et al., 2014; Sollelis et al., 2015; Zhang and Matlashewski, 2015). A combination of CRISPR/Cas9 with customary tools has resulted in a significantly efficient production of transgenic strains, as reviewed by Cui and Yu (2016) (Figure 4A). Further innovation in these organisms merits the application of even more powerful *state-of-the-art* tools and methods that have become available meanwhile. The debut of dCas9 is one such breakthrough, which has expanded the use of original CRISPR/Cas9 (Qi et al., 2013). Based on an inactive form of Cas9, it can be used to block transcription, resulting in a silencing or knockdown of the target gene (Figure 4B). It may be especially suitable for *Plasmodium* species, currently facing a dearth of efficient tools for conditional mutagenesis. Although inducible gene silencing in *Plasmodium* has been achieved, a simplified method for common application is still lacking (de Koning-Ward et al., 2015). A chemically- induced expression of dCas9 in conjunction with appropriate gRNA may provide an effective tool to this end. A bit more elegantly regulated dCas9 activation can also be accomplished by tethering it to a heat- or light-sensing motif (Richter et al., 2016). While *Toxoplasma* and *Trypanosoma* may not urgently need the dCas9-based inducible silencing, it may nonetheless be more effective, when the customary methods fail to deliver unequivocal results (Limenitakis and Soldati-Favre, 2011; Burle-Caldas Gde et al., 2015).

The feature that dCas9 can bind to a region without altering the genome has also inspired a series of other usage involving expression of dCas9 fused to a reporter/effector protein (Figure 4B). A dCas9-GFP fusion, for example, can be used for highlighting the genomic loci of interest, or if dCas9 is conjugated to an effector protein that can modify the genome, one can achieve meditated effects at the intended locus (Ma et al., 2016). We can easily envision a utility of such a system to exert specific epigenetic alterations in the parasite genomes to study remarkable phenomena of stage switching. Recently, the group of Feng Zhang has described a novel member of the Cas9 family, Cpf1 from *Francisella tularensis* (Zetsche et al., 2015). CRISPR/Cpf1 requires a shorter cr-RNA backbone with different structure for binding the gene of interest, adding further value to the system (Figure 4C). It also creates a cohesive nick after incising the distal end of the target locus, which allows multiple rounds of cleavage until desired recombination has occurred, elevating the efficiency of mutagenesis. Using cr-RNA with specific designs, researchers were able to edit a batch of genes at a time in plant and mammalian cells (Wang et al., 2017; Zetsche et al., 2017). CRISPR/Cpf1 is expected to be useful for studying proteins with redundant functions or offsetting pathways. It may also be desired for large-scale editing in parasites, as reported in *Toxoplasma gondii* using CRISPR/Cas9 (Sidik et al., 2016).

Another exciting application employs a dual-CRISPR method, which has been successfully applied in *Caenorhabditis elegans* (Chen et al., 2014). The dual-CRISPR procedure requires two gRNA binding the initial and terminal regions of the gene of interest, leading to a deletion of the entire locus (Figure 4A). It will be more suitable for the mutagenesis of long genomic fragments because the efficiency of homologous crossover can be much lower at such loci. A wider utility of the dual- CRISPR method could also resolve the limitation of transgenic selection markers in parasites. Combining it with a ± marker (hypoxanthine-xanthine-guanine phosphoribosyl transferase), an in-and-out strategy can be implemented, which will allow recycling of the selection cassette for the next round of gene editing. At the end, it is worth noting a recent study reporting an Argonaute endonuclease from *Natronobacterium gregoryi* (NgAgo), whose function is mediated by a guide-DNA instead of a guide-RNA (Gao et al., 2016); its proficiency is currently under debate however (Blow, 2016). Pending wider endorsement, it may offer a simple and economical alternative to the CRISPR system, primarily in organisms where expression of gRNA is not easily attainable.

As for those *neglected* parasites, commissioning of CRISPR technology is anticipated to break the deadlock, as shown by ingenious application of CRISPR/Cas9 in *Cryptosporidium parvum* (Vinayak et al., 2015). In essence, the machinery is wide open to all parasites, and every tool discussed here is equally applicable to them. It will however be more sensible to begin with simple gene modifications in atypical organisms before advancing further. The non-model parasites, whose genomes are not well-sequenced or annotated, NgAgo might be preferred since it does not require making of a vector for expressing gRNA under the control of the U6 elements. Ectopic expression of NgAgo along with synthetic oligonucleotides shall be sufficient to perform gene manipulation.

## Concluding remarks

Comparative genomics and molecular manipulation have proven imperative to illuminate the parasite survival, persistence, divergent lifecycle strategies, and lineage-specific adaptations. The prime challenge lying ahead now is how best to capitalize on triumphs thus far. The CRISPR-directed genome engineering has already touched on the realm of molecular parasitology, and the field is ready to receive some major upgrades. Application of customized CRISPR or CRISPR-like systems is poised to expand the efficacy of our gene-editing arsenals more than ever. It is clear that CRISPR may not offer an ultimate panacea to all problems impeding the parasite research, but it is for sure going to change the way, we will design our favorite parasites in imminent future.

## Author contributions

All authors listed have made a substantial, direct and intellectual contribution to the work, and approved it for publication.

### Conflict of interest statement

The authors declare that the research was conducted in the absence of any commercial or financial relationships that could be construed as a potential conflict of interest.
